# The Tobacco Endgame—Eradicating a Worsening Epidemic: A Joint Opinion From the American Heart Association, World Heart Federation, American College of Cardiology, and the European Society of Cardiology

**DOI:** 10.5334/gh.1061

**Published:** 2021-05-26

**Authors:** Jeffrey Willett, Stephan Achenbach, Fausto J. Pinto, Athena Poppas, Mitchell S. V. Elkind

**Affiliations:** 1 Integrated Tobacco Strategy, American Heart Association, Dallas, TX, US; 2 European Society of Cardiology, FR; 3 Department of Cardiology, Friedrich-Alexander-Universität Erlangen-Nürnberg, DE; 4 World Heart Federation, CH; 5 AIDFM, Hospital de Santa Maria, Lisboa, PT; 6 American College of Cardiology, US; 7 Brown University, Lifespan Cardiovascular Institute, Providence, RI, US; 8 American Heart Association, US; 9 Departments of Neurology and Epidemiology, Columbia University, New York, US

**Keywords:** Cardiology, tobacco

## Problem

Tobacco use continues to be a primary contributor to the global burden of disease, causing an estimated 12% of deaths worldwide among people ≥30 years of age [1]. Annually, tobacco kills 8 million people around the world, including 1.2 million nonsmokers who are exposed to secondhand smoke [2]. Globally, 21% of adults, more than one billion people, are current smokers, and >80% of the world’s smokers live in low- and middle-income countries. Most adult smokers report wanting to quit, but too many find it challenging to quit successfully. To further reduce smoking worldwide, the World Health Organization (WHO) aims to support 100 million smokers in quitting for good through the “Commit to Quit” campaign launched on World No Tobacco Day [3]. Greater global efforts are needed to build on the WHO’s goals and drive us more rapidly toward a tobacco endgame.

Despite global reductions in tobacco use [4], the introduction of electronic cigarettes and other newer tobacco products with flavorings is dramatically impacting tobacco use in youth in certain parts of the world. In Europe, rates of e-cigarette use among 13- to 15-year-olds are highest in Poland (23.4%), Ukraine (18.4%), Latvia (18.0%), and Italy (17.5%) [5]. In the United States, where government regulation of electronic cigarettes is nascent, the rate of youth use of electronic cigarettes has increased significantly. In 2020, more than 3.6 million adolescents in the United States used electronic cigarettes, including 19.6% of young people in grades 10 through 12 [6]. Heated tobacco, nicotine pouch products, and other emerging tobacco products typically include sweet, minty, and other nontobacco characterizing flavors and pose risks to global health, particularly among the young.

With this joint statement, the American Heart Association (AHA), World Heart Federation, European Society of Cardiology, and American College of Cardiology call for greater action on a global scale to end the tobacco epidemic once and for all. Governments must take more immediate action to implement the WHO’s MPOWER [7] framework, which outlines 6 essential policy strategies proven to reduce tobacco use, as contained in the WHO Framework Convention on Tobacco Control. To help accelerate progress made through WHO’s “Commit to Quit” campaign, countries must fund comprehensive tobacco prevention strategies that allow full implementation of the MPOWER framework. Furthermore, governments must effectively regulate electronic cigarettes and other emerging tobacco products to protect young people and improve public health. The Figure 1 provides a summation of the current situation and the need for action.

**Figure:**
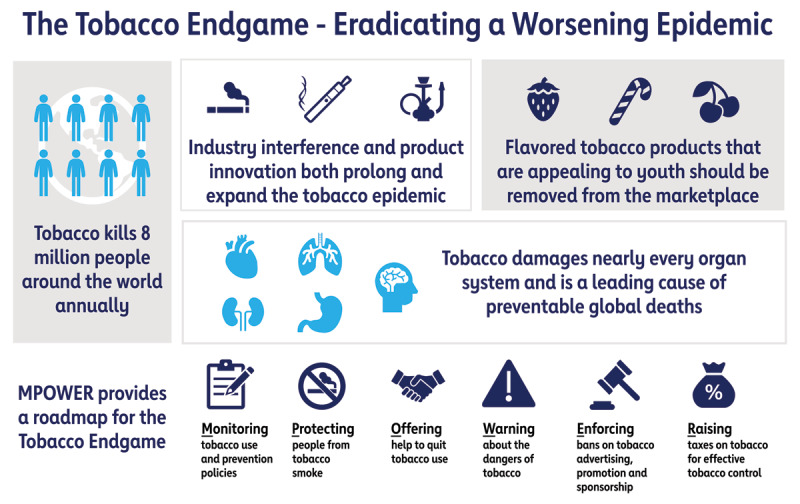
**The tobacco endgame: eradicating a worsening epidemic.** Illustration of the dangers of tobacco and the marketing innovations associated with it, as well as a proposed pathway to eradication of the tobacco epidemic.

## Nicotine, Tobacco Use, and Health

### Nicotine and Health

Although most tobacco-related morbidity and mortality is attributable to other chemicals, nicotine is the main addictive substance in tobacco products that keeps individuals using tobacco and continuing their risk for suffering tobacco-related harms. As such, the concept of nicotine addiction and its consequences, in terms of harmful tobacco product use, should be central to regulatory and policy approaches to reduce tobacco use.

Nicotine poses risks to the cardiovascular system, including causing an increase in blood pressure, heart rate, flow of blood to the heart, and a narrowing of the arteries. Nicotine may also contribute to the hardening of the arterial walls, which in turn can lead to a heart attack. Nicotine also impacts brain development and poses dangers to youth, pregnant women, and the developing fetus [8]. Nicotine exposure during adolescence can cause addiction and harm the developing brain. During pregnancy, nicotine can cross the placenta and result in multiple adverse consequences, including sudden infant death syndrome.

A greater understanding of the impacts of nicotine on cardiovascular health, and nicotine delivery products on children and youth, is necessary to inform further treatment and regulatory approaches to nicotine. Toward that end, the AHA committed $17 million [9] in early 2020 to fund research regarding the adverse health effects of nicotine and to identify more effective treatment interventions to help young people quit nicotine products.

### Combustible Cigarettes and Health

The deleterious health consequences of cigarette smoking are very well established. Smoking combustible cigarettes negatively impacts nearly every organ of the body [10], and the life expectancy of cigarette smokers is roughly 10 years less than nonsmokers [11]. Globally, smoking and exposure to secondhand smoke are responsible for roughly 1 in 5 (21%) of all deaths attributed to coronary heart disease. Tobacco use also increases risk associated with emerging diseases; as a recent example, available evidence suggests cigarette smoking is associated with increased severity of disease and death in hospitalized patients with coronavirus disease 2019 (COVID-19) [12]. Because the use of combustible tobacco products is the leading cause of preventable mortality and morbidity, substantially reducing such use represents one of the most significant opportunities to improve global health.

### Electronic Cigarettes and Health

Long-term epidemiological studies of the individual and public health impact of e-cigarettes are challenging because these products have evolved rapidly since their emergence in the mid 2000s. In its comprehensive review, the US National Academies of Science, Engineering, and Medicine stated there is “conclusive evidence that in addition to nicotine, most e-cigarette products contain and emit numerous potentially toxic substances” and that potential exposure to these toxic substances is highly variable given the range of product characteristics and how e-cigarette devices are used [13]. Compared with the use of combustible tobacco cigarettes, a very high-risk comparator, e-cigarette use likely poses less risk. However, there is growing evidence that e-cigarettes and their aerosol constituents, nicotine, vaporizing solvents, particulate matter, metals, and flavorings can have deleterious effects on the cardiovascular system [14], respiratory system [15], and brain [16].

Although the long-term health effects on adults are unclear, e-cigarette use among youth poses a clear threat to public health. In the United States, the dramatic increase in e-cigarette use among young people is unprecedented [17], and millions of young people who would never have used cigarettes or other tobacco products are using e-cigarettes. The introduction of high-nicotine delivery systems such as JUUL transformed the e-cigarette landscape and increased the addiction potential for young people. E-cigarette use is also associated with increased odds of smoking combustible cigarettes among adolescents who had no previous intention of smoking conventional cigarettes [18], belying assertions that e-cigarettes play a major role in reducing overall nicotine and tobacco use.

Evidence is limited regarding the efficacy of e-cigarettes as a smoking cessation aid [13]. Many e-cigarette users also continue to smoke cigarettes, and dual use of e-cigarettes while continuing to smoke traditional cigarettes is not associated with higher rates of quitting [19]. A 2020 Cochrane review found only moderate evidence that quit rates were higher among those randomly assigned to receive nicotine e-cigarettes compared with nicotine replacement therapy and among those randomly assigned to receive nicotine e-cigarettes compared with nonnicotine e-cigarettes [20]; however, these findings were based on only a few large randomized clinical trials. More research is needed to understand the efficacy of e-cigarettes in promoting quitting relative to approved pharmacotherapies. Additional work is also required to assess the prevalence and impact of dual use, whether the use of newer tobacco products is more likely to lead to complete abstinence from combustible tobacco use, and whether these products and e-cigarettes have adverse health effects if they are continued long-term.

### Novel Tobacco Products and Health

Heated tobacco, nicotine pouch products, and other novel tobacco products loosely represent an emerging class of tobacco products being marketed by industry as reduced-exposure or modified-risk products. There is currently limited evidence regarding either the long-term individual health risks posed by these products or their potential public health impact. Heated tobacco products contain nicotine and other potentially harmful constituents, and the potential of reduced exposure does not mean they are harmless or inherently related to reduced risk. Nicotine pouch products are one of the fastest-growing segments of the retail tobacco market in the United States, and there are strong concerns regarding their accessibility to and use by youth. Governments should introduce and enforce strong systems for thorough premarket assessment of any novel products and prevent industry from making health claims about products that are not substantiated by rigorous research and not authorized through regulatory review. Tobacco control interventions, such as MPOWER, should be applied to all tobacco products.

## The Role of Flavors

Although several countries, including the European Union, United States, and Canada, have restricted the sale of certain flavored tobacco products, greater action must be taken to ensure flavored tobacco products are not increasing tobacco-related disparities and promoting youth initiation. For instance, in Singapore, multiple menthol cigarette brands were introduced in the 1980s to appeal to young smokers, and in 2018, menthol cigarettes represented 48% of the overall market [21]. In the Philippines, where menthol cigarette use is high, cigarettes with flavor capsules have become increasingly popular [22]. Today, roughly half of all US youth who smoke cigarettes report using menthol cigarettes [23], and an estimated 86% of Black smokers and 46% of Hispanic smokers smoke menthol cigarettes compared with only 29% of White smokers. Countries should follow the lead of the European Union, which has prohibited the sale of menthol cigarettes through the EU Tobacco Products Directive. Newer tobacco products, including e-cigarettes, heated tobacco, and nicotine pouch products, are sold in a wide range of youth-appealing flavors. Greater global action must be taken to remove all youth-appealing and other nontobacco characterizing flavors from all tobacco products.

## Opportunity for Global Impact

There is tremendous potential to equitably reduce cardiovascular disease by implementing population-based tobacco prevention and control strategies. Implementing the WHO MPOWER framework effectively reduces tobacco use among adults and youth [24]. Raising the price of tobacco products, through excise taxes and other means, and youth-targeted countermarketing campaigns effectively reduce tobacco use among youth [25]. With these evidence-based strategies available, the WHO is monitoring progress toward a global target of a 30% relative reduction in tobacco use prevalence by 2025. Similarly, the AHA is working toward an ambitious tobacco endgame goal of 5% or less tobacco use prevalence in the United States [26]. Unfortunately, current rates of tobacco use suggest these goals will not be met without stronger policy development and implementation.

An estimated 780 million adult smokers worldwide want to quit [3], and the WHO’s “Commit to Quit” campaign aims to support 100 million of them in quitting successfully. Greater efforts must be made to support adult smokers who want to quit through treatment interventions and through policy measures that create supportive environments for cessation.

Evidence is clear that e-cigarettes pose a significant global health threat through dramatic increases in youth tobacco and nicotine use. Many countries have banned the commercialization of e-cigarettes and heated tobacco products using the precautionary principle (ie, that the introduction of a new product or process whose ultimate effects are unknown should be deferred until scientific evidence of its safety is available) as justification. The International Union Against Tuberculosis and Lung Disease has outlined a strong rationale for why low- and middle-income countries should ban the sale of all electronic nicotine delivery systems entirely [27]. Globally, more effective regulation of online sales of e-cigarettes and e-liquids is needed to make e-cigarettes less accessible to youth. Actions must be taken quickly in countries where e-cigarettes are widely used if we are to reduce youth nicotine addiction and tobacco use while providing access to comprehensive, evidence-based cessation services as a safer alternative for adults who wish to quit smoking combustible cigarettes.

## Addressing the Challenge: The Role of AHA and Its Partners

The AHA, World Heart Federation, American College of Cardiology, and European Society of Cardiology are committed to ending the global tobacco epidemic through advocacy for policies proven to reduce tobacco use and by encouraging bolder government actions to protect public health. Together and with other global partners, we continue to **monitor and draw attention to tobacco industry practices** that promote its addictive and deadly products. Recognizing the tremendous toll that combustible tobacco products have on global health, we call for stronger government actions that **more rapidly reduce the use of combustible tobacco products**. Toward this aim, we support **lowering nicotine concentrations in all combustible tobacco products** while ensuring the next generation is not addicted to new nicotine delivery products.

The MPOWER framework and decades of experience in tobacco control provide a road map to achieving the tobacco endgame. We are calling on governments to **raise the price of tobacco products** to levels that effectively promote adult cessation and substantially reduce youth initiation. Eliminating the sale of menthol cigarettes and other flavored tobacco products is essential to eradicating the global tobacco epidemic, and governments should **eliminate the sale of all tobacco products with characterizing flavors** to substantially reduce youth initiation and address tobacco-related disparities. Governments should **protect people from tobacco smoke** by enacting comprehensive smoke-free policies, including combustible, heated tobacco, and electronic products, for all indoor public places. To the extent possible under existing legal frameworks, governments should **establish and enforce comprehensive bans on tobacco industry advertising, promotions, and sponsorships**. Effective pictorial health warnings should be included on all tobacco product packaging, and where possible under existing legal frameworks, plain/standardized tobacco product packaging should be used.

We will also advocate for **additional and more robust research** to identify additional strategies that help established tobacco users quit successfully, with a particular focus on youth. We expect the same rigor of research and regulation to be applied to all products claiming efficacy in helping adults quit. For instance, well-designed clinical trials of any products with cessation potential, such as e-cigarettes, must be conducted under and reviewed by relevant drug administration laws and agencies.

Finally, governments must take greater actions to **restrict or prohibit the sale of tobacco products** while ensuring established tobacco users have the support needed to quit successfully.
